# Performance of Wisconsin Card Sorting Test in five-year-old children in Taiwan: Relationship to intelligence and cognitive development

**DOI:** 10.1371/journal.pone.0202099

**Published:** 2018-08-30

**Authors:** For-Wey Lung, Po-Fei Chen, Bih-Ching Shu

**Affiliations:** 1 Calo Psychiatric Center, Pingtung County, Xinpi Township, Taiwan; 2 Graduate Institute of Medical Science, National Defense Medical Center, Taipei City, Taiwan; 3 Institute of Allied Health Sciences and Department of Nursing, National Cheng Kung University, Tainan City, Taiwan; University of Windsor, CANADA

## Abstract

**Objective:**

(1) To develop the norm of Wisconsin Card Sorting Test (WCST), (2) to investigate the pathway of the indices for WCST performances and (3) the association between WCST, intelligence quotient (IQ), and parent-report measures of children’s development in typically developing five-year-olds in the community.

**Method:**

Fifty-three children were recruited from community health centers. The WCST, Wechsler Preschool and Primary Scale of Intelligence-Revised (WPPSI-R), and Taiwan Birth Cohort Study- Developmental Instrument (TBCS-DI) was used to measure children's executive function, IQ and parent report of children's development respectively.

**Results:**

Mean categories achieved (CA) was 2.02 (standard deviation [SD] = 1.41), and percent conceptual level response (PCLR) was 29.85(SD = 18.36) in five year-olds. The WCST indices showed a pathway relationship of PCLR being negatively associated with perseverative error (PE), and PE and non- perseverative error being negatively associated with CA. Association among the PCLR index of the WCST, cognitive domain of the TBCS-DI, and performance IQ and verbal IQ of the WPPSI-R was found.

**Conclusion:**

Regular improvement with age was found compared to the norm of six-year-olds in a previous study of children from the same region. The number sorting criteria was more difficult thus they continued to perform persistent errors of color or form when sorting. Association was found among the professional administered IQ, computerized WCST, and a parent report developmental instrument. Showing parent report is an accurate reflection of children's cognitive development at this age.

## Introduction

The Wisconsin Card Sorting Test (WCST) was first introduced as a measure of abstract reasoning and the ability to shift cognitive strategies in response to changing environmental contingencies [1], and has become a well-established measure of executive function [2]. Executive function refers to a set of cognitive skills, and influences future academic and behavioral competencies [3]. For the ability to form and flexibly change concepts is vital in the development of academic skills [4]. By trial and error with feedback, participants need to find a relevant sorting rule out of three possible sorting rules (color, shape, or number). The sorting rule changes without warning after ten correct sorts, requiring participants to find the new sorting rule. Although initially designed for adults, its use recently has been expanded to include children [5], with developmental norms for children as young as six-year-olds being constructed in Taiwan [6], and as young as five years old in the United States [7]. Adult level of performance is reached at the age of ten [8, 9]. Although the application of the WCST in children have been studied in previous described studies, however, the number of literature is scarce, therefore the norm and validity of its use in children still need more investigation.

Although executive function is correlated with education in elders [10], however, its association with intelligence quotient (IQ) have shown inconsistent results [7, 10–13]. Welsh, Pennington and Groisser [7] found no correlation between WCST and IQ in typically developing children from six- to 12-year olds. Ardila, Galeano, and Rosselli [11] also found no correlation between WCST and IQ in college students. However, Riccio et al. [13] found correlation between the performance IQ of Wechsler Intelligence Scale for Children- Revised (WISC-R) and the WCST in children between nine-years and nine-years 11 months with attention deficit hyperactivity disorder. Additionally, Arffa et al. [12] found the WCST performance of above average children (IQ>130) clearly improved in WCST perseverative errors, nonperseverative errors, total errors and trials to complete category compared to children with average intelligence. In adolescents, Ardila et al. [11] also found a correlation between the WCST and the Verbal Intelligence Quotient (VIQ) of the WISC-R, but not the Performance Intelligence Quotient (PIQ). In adults, Chien, Huang and Lung [14] found that the WCST can be used as the first-stage screening instrument for intellectual disability in adults. Since the association between executive function (measured using the WCST) and IQ have shown inconsistent results in previous research, the relationship between the WCST and IQ in young children need to be investigated.

A previous study found that developmental disorders in general include deficits in executive function [15]. Zhu, Tang and Shi [16] compared children with developmental coordination disorder and typically developing children, and found children with developmental coordination disorder performed with more errors, more perseverative responses and more perseverative errors in the WCST than the controls. However, the association of children's development in the community with WCST performances have rarely been investigated.

Early identification of developmental delay is important, since early intervention can lead to a better outcome for the child, a lesser burden on the family, and less financial expenditure by society in the long run [17]. Thus the norm of the WCST for preschool children to be used for screening for executive function and its association with IQ and children's development need to be investigated. Therefore, the aim of this study was to (1) develop the norm of WCST in 5-year-olds, (2) investigate the pathway of the indices for WCST performances, and (3) the association between WCST, IQ, and parent-report measures of children’s development in typically developing five-year-olds in the community.

## Materials and methods

### Participants

Children were consecutively recruited from community health centers in Southern Taiwan; those born between July 2004 and March 2005 and registered for immunity shots were invited to participate. A total of 221 children were invited, with 100 agreeing to participate. Four stages of developmental data were collected: when children were six-months, 1.6-, three-, and five-years of age. The 5-year-old dataset was used in this study, which included assessment of children's WCST, Wechsler Preschool and Primary Scale of Intelligence-Revised (WPPSI-R), and Taiwan Birth Cohort Study- Developmental Instrument (TBCS-DI). At this stage, 53 children from the initial 100 agreed to participate and the main caregivers of these children gave their informed consent for the study. The protocol and research design was approved by the Institutional Review Board of Taipei City Hospital, Taipei, Taiwan, and is in compliance with the Helsinki Declaration.

### Materials

All children were assessed using the WCST, the WPPSI-R, and parents had to fill out the TBCS-DI. The computerized version of the WCST was used to evaluate children’s executive function, the WPPSI-R (Taiwanese version) was used to evaluate the children’s intellectual functioning, and the TBCS-DI for 5-year-olds was used to evaluate the parent-report of children’s development.

The computerized version of the WCST was administered. This computerized version with four depict figures of keyboard was developed based on the standardized criteria of Heaton, Chelune, Talley, Kay and Curtiss [18] by Tien et al. [19]. The norm for this test has been developed for six- to 11-year-old children in Taiwan [4], thus showing it can be used to test the executive function of children. According to Heaton et al.’s criteria [18], response results included categories achieved, perseverative errors and non-perseverative errors, percent of total errors, trials to complete the first category, and percent conceptual level response indices. Children were encouraged throughout the assessment, for the standardization of the study, all children completed 128 trials.

The Taiwanese version of the WPPSI-R was translated and modified from the original WPPSI-R [20] to suit the Taiwanese language and culture, and was designed test the intelligence of children between three and seven years and three months old [21]. A Taiwanese norm has been developed, and the results of the WPPSI-R are presented in three standardized, norm-referenced quotients: the VIQ, the PIQ, and the Full Scale Intelligence Quotient (FSIQ), representing the intellectual functioning of children in verbal and performance cognitive domains, and child’s general intellectual ability. The mean for these quotients is 100 (standard deviation = 15), with scores lower than 2 standard deviations from the mean considered atypical.

The TBCS-DI is a culturally-sensitive parent-report developmental instrument, with easily comprehendible items measuring children’s development in the four dimensions of gross motor, fine motor, language, and social development at 6, 18, 36, and 60 months. The 60 months scale was used in this study. The four dimensions can be separated into two domains: motor and cognitive development, with gross and fine motor combining to form the motor domain, and with language and social dimensions forming the cognitive domain. The reliability and validity of the TBCS-DI were tested in the Taiwan Birth Cohort Study, which collected developmental data for 21,248 children from birth to 5 years of age; all the scales has exhibited high reliability, internal consistency, and validity [22, 23], with higher scores implying better development.

### Statistical analysis

Data was analyzed using SPSS 17.0 (SPSS, Chicago, IL, USA) and AMOS 7.0 (SPSS, Chicago, IL, USA) software packages for Windows. Descriptive and correlation analysis was processed using SPSS 17.0 and structural equation modeling (SEM) was carried out using AMOS 7.0.

With the demographic factors controlled, SEM was used to investigate the pathway relationship among the WPPSI-R, the TBCS-DI, and the WCST. SEM uses the chi-square fit test to investigate the overall fit of the model; models resulting in non-significant chi-square of *p* value greater than 0.05, goodness-of-fit greater than 0.9, and root mean square error of approximation less than 0.1 [24] indicate that the model adequately describes the observed data. Only parsimonious models were presented, meaning that only the statistically significant variables were shown.

## Results

Of the 53 children who participated, 29 (54.7%) were male. The average age of the mothers was 29.30 (SD = 4.63) and the average age of the fathers was 33.26 (SD = 4.84). The demographic distribution and parental characteristics are shown in Table 1. Furthermore, the WCST mean and standard deviation of these five-year-olds children in the community were also shown in Table 1.

**Table 1:** Demographic distribution of the children and their parents (N = 53).

Variables	n (%)
Male	29 (54.7)
Maternal level of education	
Elementary	2 (3.8)
Middle school	2 (3.8)
High school	18 (34.0)
Associate degree	15 (28.3)
Bachelor’s degree	13 (24.5)
Graduate school	3 (5.7)
Paternal level of education	
Middle school	1 (1.9)
High school	21 (39.6)
Associate degree	12 (22.6)
Bachelor’s degree	14 (26.4)
Graduate school	5 (9.4)
Variable (range)	Mean (SD)
Parental age	
Maternal age (range: 20~44)	29.30 (4.63)
Paternal age (range: 24~45)	33.26 (4.84)
WPPSI-R performance	
PIQ (range: 66~138)	100.19 (15.70)
VIP (range: 68~124)	96.00 (13.50)
FSIQ (range: 65~124)	97.68 (13.75)
WCST performance	
Categories achieved (range: 0~6)	2.02 (1.41)
Non-perseverative errors (range: 0~63)	22.82(15.33)
Perseverative errors (range: 0~74)	30.52(19.32)
Percent conceptual level response (range: 2~66)	29.85(18.36)
Percent total errors (range: 11~99)	53.34(19.16)

WPPSI-R: Wechsler Preschool and Primary Scale of Intelligence-Revised; PIQ: performance intelligence quotient; VIQ: verbal intelligence quotient; FSIQ: full scale intelligence quotient

**Fig 1:**
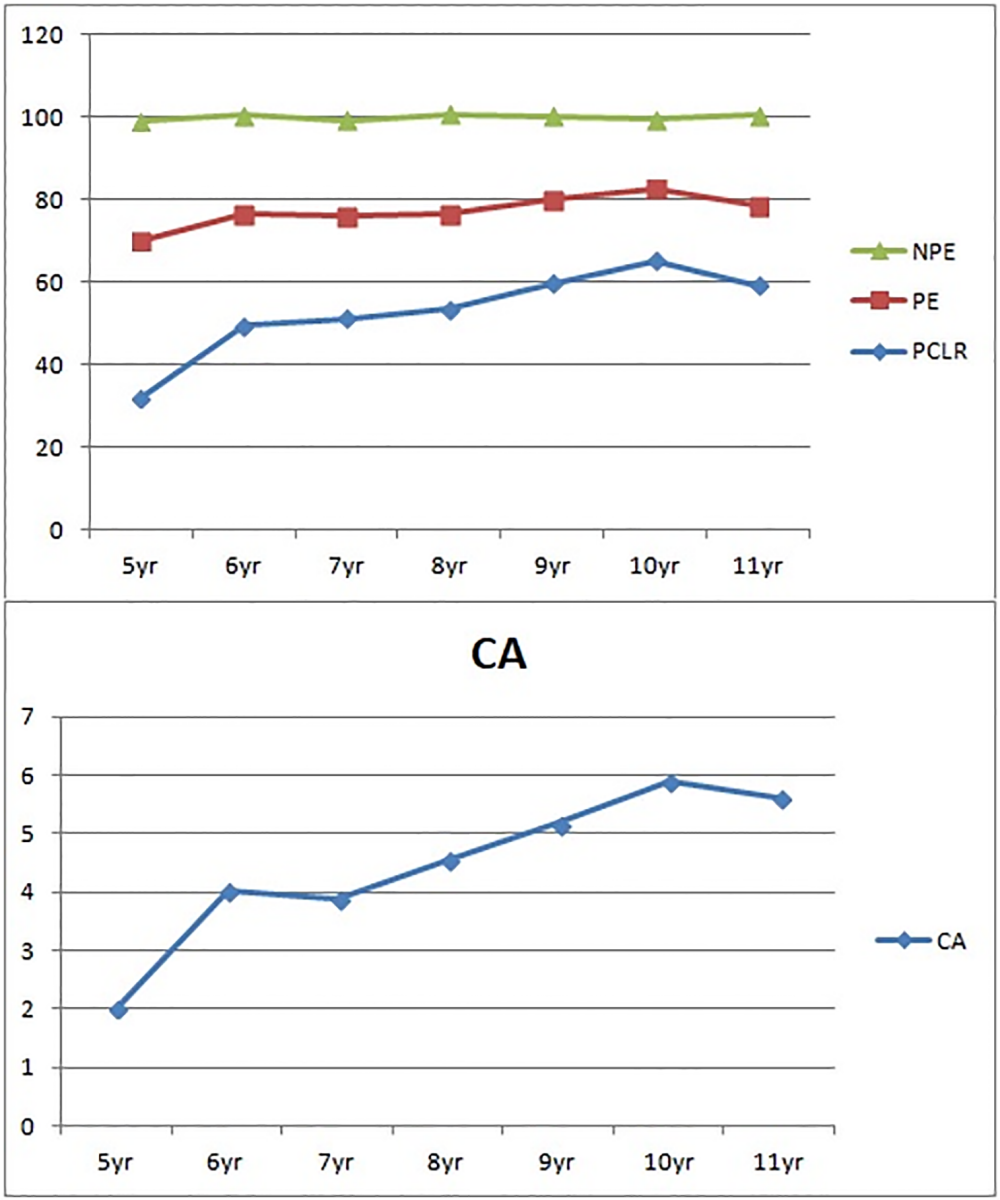
Mean score of the indices in WCST for five- to 11-year-old children. CA: Categories achieved; NPE: Non-perseverative errors; PE: Perseverative errors; PCLR: Percent conceptual level response. Data for six-to 11-year-old children from Shu, Tien, Lung, & Chang (2000).

Fig 1 shows the trajectory of children's WCST performance from five- to 11-years in the categories achieved, non-perseverative errors, perseverative errors and percent conceptual level response indices. The norm for six to 11 was from the results in Shu et al.'s [4] study. Regular improvement with age was shown, demonstrating the validity the use of WCST in five-year-olds.

The associations among the cognitive dimension of the TBCS-DI, indices of the WCST and the IQs of the WPPSI-R is shown in Table 2. Pearson correlation results showed that the cognitive domain of the TBCS-DI correlated with the percent conceptual level response and percent of total errors of the WCST (r = 0.42, p = .011; r = -0.36, p = .008). In addition, TBCS-DI was also associated with the VIQ and FSIQ of the WPPSI-R (r = 0.29, p = .037; r = 0.31, p = .024). The trials to complete the first category, categories achieved, perseverative errors, and percent conceptual level response indices of the WCST showed significant correlation with the PIQ and FSIQ of the WPPSI-R (r = -0.36, p = .033; r = -0.34, p = .046; r = 0.55, p = .001; r = 0.43, p = .008; r = -0.38, p = .023; r = -0.33, p = .048; r = 0.53, p = .001; r = 0.45, p = .006). Furthermore, the percent of total errors index of the WCST also had significant correlation with the FSIQ of the WPPSI-R (r = -0.27, p = .051). Thus showing there were association among children's cognitive development, executive function and IQ.

**Table 2:** Correlation among the cognitive dimension of the Taiwan Birth Cohort Study- Developmental Instrument (TBCS-DI), indices of the Wisconsin Card Sorting Test (WCST) and the intelligence quotient (IQ) of the Wechsler Preschool and Primary Scale of Intelligence-Revised (WPPSI-R).

	TBCS-DI	WCST TCFC	WCST CA	WCST NPE	WCST PE	WCST PCLR	WCST PTE	WPPSI PIQ	WPPSI VIQ	WPPSI FSIQ
TBCS-DI	-	-0.11	0.27	-0.17	-0.18	0.42*	-0.36**	0.26	0.29*	0.31*
WCST TCFC	-	-	-0.50**	0.14	0.12	-0.30	0.30	-0.36*	-0.17	-0.34*
WCST CA	-	-	-	-0.30	-0.39*	0.80**	-0.78**	0.55**	0.15	0.43**
WCST NPE	-	-	-	-	-0.62**	-0.29	0.28	-0.05	0.04	-0.02
WCST PE	-	-	-	-	-	-0.56**	0.58**	-0.38*	-0.18	-0.33*
WCST PCLR	-	-	-	-	-	-	-0.99**	0.53**	0.20	0.45**
WCST PTE	-	-	-	-	-	-	-	-0.24	-0.23	-0.27
WPPSI PIQ	-	-	-	-	-	-	-	-	0.44**	0.85**
WPPSI VIQ	-	-	-	-	-	-	-	-	-	0.84**

**p < .005;

*p < .001

TCFC: Trials to complete the first category; CA: Categories achieved; NPE: Non-perseverative errors; PE: Perseverative errors; PCLR: Percent conceptual level response; PTE: Percent of total errors; FSIQ: Full Scale IQ; PIQ: Performance IQ; VIQ: Verbal IQ

**Fig 2:**
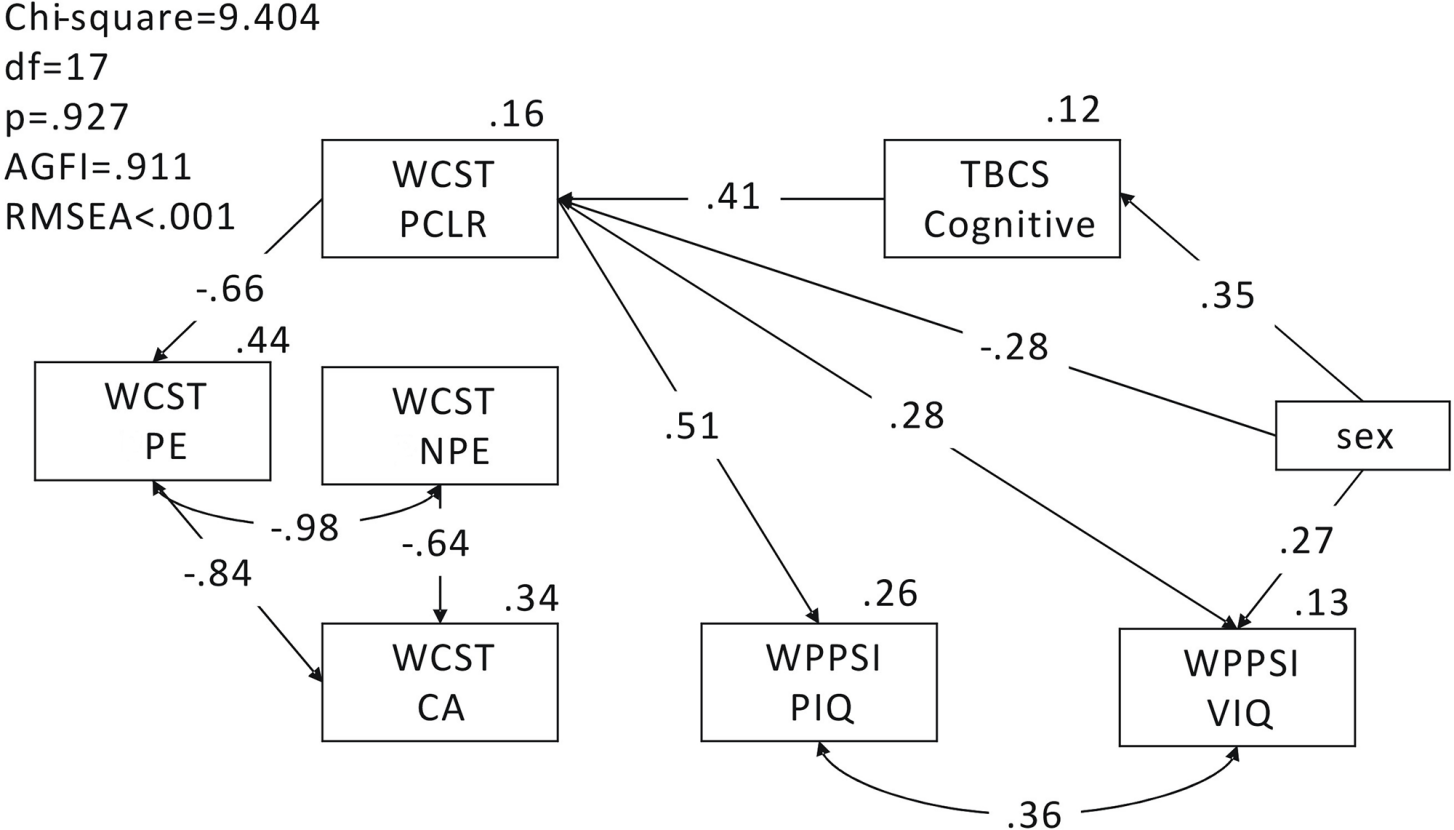
The pathway relationship among the Taiwan Birth Cohort Study (TBCS) developmental instrument, Wisconsin Card Sorting Test (WCST), and the Wechsler Preschool and Primary Scale of Intelligence (WPPSI-R). AGFI: adjusted goodness-of-fit index; RMSEA: root mean square error of approximation; CA: Categories achieved; NPE: Non-perseverative errors; PE: Perseverative errors; PCLR: Percent conceptual level response; PIQ: Performance IQ; VIQ: Verbal IQ.

The structural equation model investigated the pathway relationship among the TBCS-DI, the indices of WCST and the PIQ and VIQ of the WPPSI-R. The model resulted in a good fit, with a *p* value of 0.927 (greater than 0.05), AGFI of 0.911 (greater than 0.9) and RMSEA of less than 0.001 (less than 0.1), as shown in Fig 2. The TBCS-DI cognitive dimension was associated with the percent conceptual level response index of the WCST (β = 0.41, p = 0.003), and the percent conceptual level response index of the WCST was associated with the PIQ and VIQ of the WPPSI-R (β = 0.51, p<0.001; β = 0.28, p = 0.029). Gender differences was found in the TBCS-DI cognitive dimension, percent conceptual level response of the WCST and the VIQ of WPPSI-R, with girls performing better than boys in the cognitive dimension of TBCS-DI (β = 0.35, p = 0.007) and the VIQ of WPPSI-R (β = 0.27, p = 0.029). But boys performing better than girls in the percent conceptual level response index of WCST (β = -0.28, p = 0.037). The SEM results further validate the association among children's cognitive development, executive function and IQ, and the pathway relationship among these assessments.

Also in Fig 2, the WCST indices showed a construct of percent conceptual level response showing negative association with the perseverative errors index (β = = -0.66, p<0.001), and perseverative errors and nonperseverative errors showing negative association with the categories achieved index (β = -0.84, p<0.001; β = -0.64, p<0.001).

## Discussion

Our study found the norm of five-year-old children in the community in Taiwan, all children completed 128 trials, resulting in a mean categories achieved of 2.02 (SD = 1.41), and percent conceptual level response of 29.85(SD = 18.36). The WCST performances of these five-year-olds showed a pathway relationship of percent conceptual level response being negatively associated with perseverative errors, and perseverative errors and non-perseverative errors being negatively associated with categories achieved. Furthermore, association among the percent conceptual level response index of the WCST, cognitive domain of the parent report developmental instrument, and PIQ and VIQ of the WPPSI-R was found. These three assessments are distinctly different psychological measures of children’s cognitive development. The TBCS-DI is a parent-report developmental instrument for daily observations of children, while the WCST and the WPPSI-R are both professionally-assessed instruments. However, the WCST is a computerized, non-verbal, culturally-independent measurement of executive function, while the WPPSI-R is a more detailed assessment, measuring more aspects of a child’s intelligent quotient. Interestingly, although TBCS-DI, WCST, and WPPSI-R assess different aspect of cognitive development through different methods, association was found among the three instruments in our five-year-old community sample.

Overall, the norm of six-year-olds in Shu et al.'s study [4] showed regular improvement with age compared to the five-years-old sample in our study, as shown in Fig 1, demonstrating their validity. Furthermore, the participants recruited in this study were of the same region as that in Shu et al.'s study [4]. This result support previous normative studies showing children make rapid gains in the number of categories achieved [4, 5]. Somsen [25] also found a linear increase in the number of categories completed, and decrease in the number of errors with age, until the age of 11, after which this number reaches a plateau.

Association between children's cognitive development, executive function and children's IQ was found. More specifically, parent report of children's cognitive development was found to be associated with percent conceptual level response of the WCST, which was associated with the PIQ and VIQ of these five-year-old children. The percent conceptual level response index of the WCST was associated with the cognitive domain of the TBCS-DI, and the VIQ and PIQ of the WPPSI-R. This is in line with previous studies which found intelligence predicted more than 19% of the variance in percent conceptual level response for children ages nine to 11 years old [26]. Although previous studies have found inconsistent results in the relationship between the performance of WCST and IQ [7, 11–13]. However, Bujoreanu and Willis [27] found six-year-olds were affected by the order of administering the number sorting criteria, thus showing the number sorting criteria in the WCST measures more sophisticated cognitive function in young children, and not just the set shifting executive function ability which the WCST originally intended to measure for older children and adults. Arffa et al. [12] also found association of WCST and IQ in children higher than average IQ, and hypothesized that the WCST may measure higher level conceptual function in younger children, which is not needed for adults when completing the WCST testing.

The WCST indices showed a pathway relationship of percent conceptual level response being negatively associated with perseverative errors, and perseverative errors and non-perseverative errors being negatively associated with categories achieved. The five-year-olds in our study had difficulty sorting the number criteria, thus continued to use the color and shaping sorting criteria which caused perseverative errors to be associated with percent conceptual level response, but not non-perseverative errors. Previous studies have shown that children develop the ability of grouping concept between the age of three and four, however, the ability to spontaneously generate categories more complex than the basic color and shape continue to develop after the age of seven [28]. Bujoreanu and Willis [27] found the number criterion to be the most difficult for six-year-old children compared to color and shape, and only less than two-thirds of the participants were able to complete all three categories compared to nearly all participants in the older age groups (11–12 year-olds and 18–19 year-olds) completed all three sorting categories.

Of the demographic factors investigated, sex was the only factor which affected children’s VIQ performance, percent conceptual level response index in the WCST, and the parent-report of cognitive development, with females performed bettered than males in the cognitive domain of the TBCS-DI and VIQ at five years of age. These gender differences in children’s cognitive development have been found in previous studies, which consistently have shown females developing better verbally than males [23, 29].

A limitation of our study is the small sample size, therefore the norm and association among children's executive function, development and IQ need to be verified in a larger sample. In addition, the children were consecutively rather than randomly sampled. Since this is a community-based study, and several hours are required to complete all three instruments, not all parents were able to participate. However, our sample reflected the performance of children in the community, with 1 of 53 children scored lower than 70 in the FSIQ of the WPPSI-R (indicating intellectual disability), and 11 of 53 scored lower than 85 (indicating borderline intellectual disability). Furthermore, correlation and structural equation modeling showed consistent results.

In these community sampled five-year- olds, the WCST performance showed mean of 2.02 (SD = 1.41) for categories achieved, and a mean of 29.85(SD = 18.36) for percent conceptual level response, which could be used as a reference for the norms of five year old children in Taiwan. Furthermore, the norm of the six-year- olds in Shu et al.'s study [4] showed improvement compared to the sample of five-year- olds in the same region. Association was found among three different instrument, the professional administered WPPSI-R measuring IQ, computerized WCST measuring executive function, and a parent report developmental instrument, showing association among the cognitive development, IQ and executive function at this age. Furthermore, parent report is an accurate reflection of children's cognitive development at this age. A larger scale study may be needed to verify the results of our investigation.

## Supporting information

S1 Dataset (SAV)
